# Supplementary material for: MetaRibo-Seq measures translation in microbiomes
Source: Nat Commun. 2020 Jun 29;11:3268. doi: 10.1038/s41467-020-17081-z (PMC7324362; doi:10.1038/s41467-020-17081-z)
Supplement: Supplementary file 10 — Supplementary Data 7 [file 41467_2020_17081_MOESM10_ESM.zip › File2/Confidence_High_Taxonomy/240789_out.krona.html]

Javascript must be enabled to view this page.

members
magnitude
magnitudeUnassigned
count
unassigned
taxon
rank

240789\_out

17

superkingdom
2
17

17
1239
phylum

186801
class
17

186802
order
17

family
186803
17

841
genus

SRS011084\_contig\_number\_contig-100\_287.214027SRS019267\_contig\_number\_contig-100\_17257.17257SRS048164\_contig\_number\_30345SRS056537\_contig\_number\_25893SRS058070\_contig\_number\_31914SRS058723\_contig\_number\_contig-100\_11898.11898SRS144297\_contig\_number\_contig-100\_5104.111975SRS149784\_contig\_number\_contig-100\_23596.23597
17
8

1

SRS052027\_contig\_number\_12864
species
2049040

8
species

SRS013951\_contig\_number\_26506SRS014459\_contig\_number\_28200SRS014923\_contig\_number\_26863SRS015854\_contig\_number\_14217SRS016954\_contig\_number\_26570SRS019397\_contig\_number\_13223SRS075021\_contig\_number\_contig-100\_112.60709SRS144714\_contig\_number\_contig-100\_151.172533
360807
